# Early-career general practitioners’ antibiotic prescribing for acute infections: a systematic review

**DOI:** 10.1093/jac/dkae002

**Published:** 2024-01-22

**Authors:** Emma J Baillie, Greg Merlo, Mieke L Van Driel, Parker J Magin, Lisa Hall

**Affiliations:** General Practice Clinical Unit, Faculty of Medicine, The University of Queensland, Brisbane, QLD, Australia; General Practice Clinical Unit, Faculty of Medicine, The University of Queensland, Brisbane, QLD, Australia; General Practice Clinical Unit, Faculty of Medicine, The University of Queensland, Brisbane, QLD, Australia; School of Medicine and Public Health, University of Newcastle, Callaghan, NSW, Australia; GP Training Research Department, Royal Australian College of General Practitioners, Callaghan, NSW, Australia; School of Public Health, The University of Queensland, Brisbane, QLD, Australia

## Abstract

**Background:**

Antimicrobial resistance is a worldwide threat, exacerbated by inappropriate prescribing. Most antibiotic prescribing occurs in primary care. Early-career GPs are important for the future of antibiotic prescribing and curbing antimicrobial resistance.

**Objectives:**

To determine antibiotic prescribing patterns by early-career GPs for common acute infections.

**Methods:**

A systematic literature search was conducted using PubMed, Embase and Scopus. Two authors independently screened abstracts and full texts for inclusion. Primary outcomes were antibiotic prescribing rates for common acute infections by GPs with experience of 10 years or less. Secondary outcomes were any associations between working experience and antibiotic prescribing.

**Results:**

Of 1483 records retrieved, we identified 41 relevant studies. Early-career GPs were less likely to prescribe antibiotics compared with their more experienced colleagues (OR range 0.23–0.67). Their antibiotic prescribing rates for ‘any respiratory condition’ ranged from 14.6% to 52%, and for upper respiratory tract infections from 13.5% to 33%. Prescribing for acute bronchitis varied by country, from 15.9% in Sweden to 26% in the USA and 63%–73% in Australia. Condition-specific data for all other included acute infections, such as sinusitis and acute otitis media, were limited to the Australian context.

**Conclusions:**

Early-career GPs prescribe fewer antibiotics than later-career GPs. However, there are still significant improvements to be made for common acute conditions, as their prescribing is higher than recommended benchmarks. Addressing antimicrobial resistance requires an ongoing worldwide effort and early-career GPs should be the target for long-term change.

## Introduction

Antimicrobial resistance is growing worldwide, exacerbated by inappropriate antibiotic prescribing.^1,2^ The majority of human antibiotic use occurs in general practice.^3,4^ One of the most significant determinants of antibiotic prescribing is the prescribing habits of individual GPs.^5,6^ These prescribing habits are likely formed during training and the early years of their careers.^7–9^ Evidence suggests that once prescribing habits are formed, they tend to remain stable over time.^8,9^ Training of GPs varies between countries, although typically following a similar process across nations. As part of the medical school curriculum and in some countries after graduating, doctors spend a few years in hospital before progressing to specialty vocational training, including general practice.^10–12^ A few countries do not have mandatory specialist general practice training, allowing graduates to start working as a GP straight after graduation.^13^ Research has shown that medical students consider resistance a public health concern, yet still have serious information gaps with regard to antibiotic usage.^14^ A qualitative study by Dallas *et al.*^15^ found that GPs in vocational training in Australia are ‘used to’ prescribing antibiotics in the hospital setting where they regularly see serious infections.^15^ The transition from the hospital setting to general practice is a crucial point in a clinician’s career.^16^ Therefore, investigating the antibiotic prescribing of GPs during the early-career period may inform the future stewardship of antimicrobial prescribing.

This group may be more receptive to interventions, given they have not yet formed these long-term prescribing habits. A systematic review examining interventions in junior doctors and medical students demonstrated prescribing behaviours can be altered.^17^

Despite the importance of a GP’s experiences early in their career for determining their ongoing antibiotic prescribing behaviour, there are currently no systematic reviews of studies on this topic.

We aimed to explore the antibiotic prescribing patterns of early-career GPs for acute infections, and if there is a relationship between antibiotic prescribing and working experience.

## Methods

This systematic review was registered with PROSPERO (CRD42021273935) and follows Preferred Reporting Items for Systematic Reviews and Meta-Analyses (PRISMA) guidelines.^18^

## Eligibility criteria

Studies were included if they met the following criteria: (i) setting in general practice/family medicine; (ii) examined early-career GPs (defined below) and/or examined the influence of work experience (or equivalent variable); (iii) observational studies or control arms of randomized controlled studies; (iv) examined overall ‘antibiotic prescribing’ and/or prescribing for common acute infections (defined below).

## Key terms and definitions

We defined early career as the first 10 years in the profession post-graduation, consistent with published literature.^19–21^ ‘Early career’ terminology differs across countries, commonly used are terms that describe general practice specialty training—trainees, ‘registrars’ (Australia, Hong Kong and UK) or ‘residents’ (Americas, Europe).

‘Common acute infections’ were defined as self-limiting mild infections or where antibiotics are generally not indicated. Classes of infections included: upper respiratory tract infections (URTIs), lower respiratory tract infections (LRTIs), gastrointestinal infections (GITs), urinary tract infections (UTIs) and skin/soft tissue infections (SSTIs).

‘Antibiotic prescribing’ included any drug formulations or administration modalities, and prescribing for patients of any age.

## Exclusion criteria

Studies were excluded if they were: (i) examining complex or severe conditions, e.g. COPD, chronic bronchitis, community-acquired pneumonia, recurrent infection or severe infection; (ii) examining prescribing in complex patients, e.g. immune compromised, UTIs in males, or pregnant women; (iii) in non-general practice settings in primary care, e.g. residential aged care facilities, emergency departments and urgent care; (iv) prescribers who are not GPs but work in primary care, e.g. paediatricians or nurse practitioners; or (v) studies with fewer than five early-career GPs.

## Search strategy

Databases searched were PubMed, Embase and Scopus. Articles were included if they were original research, and no limitations were placed on publication date. The last search date was 17 October 2022.

Articles not in English were examined separately and translated to English via Google Translate. Included full-text articles were then searched manually for additional records via citation searching, using Google Scholar.

## Example search (PubMed)

Search terms used were ‘primary care’ OR ‘general practice’ OR ‘general practitioner’ OR ‘family medicine’ OR ‘family practice’ OR ‘community care’ AND ‘early-career’ OR ‘trainee’ OR ‘registrar’ OR ‘resident’ OR ‘student’ OR ‘vocation’ AND ‘antibiotic’ OR ‘antibacterial agent’ OR ‘antibiotic resistance’ OR ‘anti-infective agent’ OR ‘antimicrobial stewardship’ OR ‘resistance’ OR ‘antimicrobials’. See Table S1 (available as Supplementary data at *JAC* Online) for our full search strategy.

## Outcomes

Primary outcomes were antibiotic prescribing rates for common acute infections by early-career GPs. Secondary outcomes were any associations between working experience of the GP and antibiotic prescribing.

## Data collection

Search results were downloaded into Covidence, and duplicates removed. Two authors (E.J.B. and G.M.) screened titles, abstracts and full texts independently using Covidence. Disagreements were discussed and, if required, resolved by a third author (M.L.V.D.). Data extracted into an Excel spreadsheet included study characteristics (setting, design, country), GP demographics (age, number, definition of early career, sex), patient population (age, number) and outcomes (condition, prescribing rates, working experience variable, statistical measures).

## Data analysis

Data were tabulated and narrative analysis was undertaken. Subgroup synthesis of primary outcomes was by condition, with prescribing rates and 95% CIs where available. Secondary outcomes were presented by the nature of the relationship (direction and magnitude) between prescribing and experience, and variable used.

## Risk of bias in individual studies

The Newcastle–Ottawa scale was used for cohort and case–control studies, and adapted for cross-sectional studies; see Table S2.^22^ Control arms of randomized controlled trials (RCTs) were assessed using the Cochrane Risk of Bias tool.^23^ E.J.B. performed the risk of bias and G.M. checked a randomly selected number of studies.

## Ethics

Ethical approval was not required.

## Results

### Search results and study characteristics

**Figure 1.**
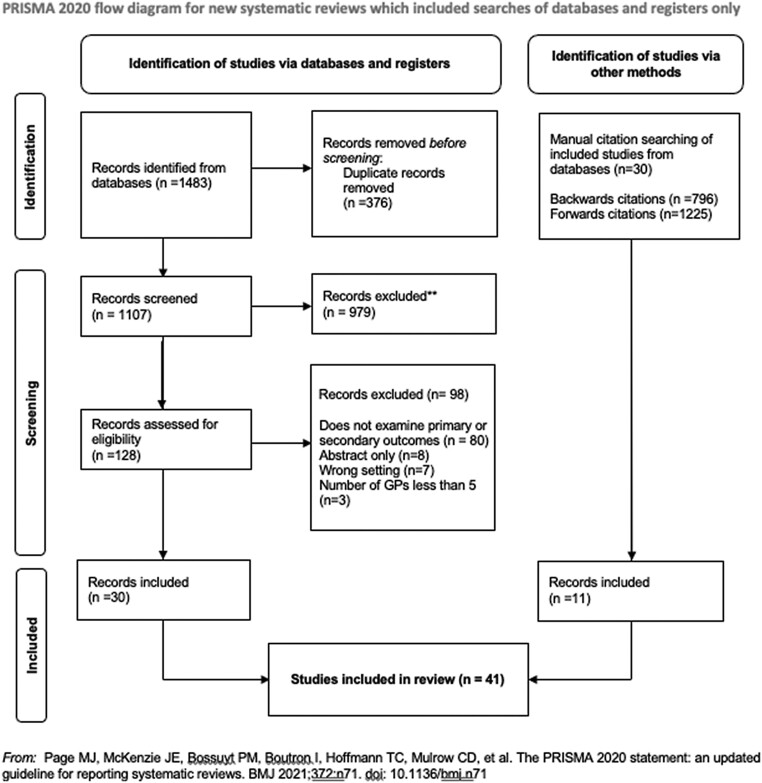
Early-career GPs’ antibiotic prescribing: a systematic review—PRISMA diagram.

Of the 1483 records identified, 376 duplicates were removed, leaving 1107 records for title and abstract screening. There were 128 records eligible for full-text screening; see PRISMA diagram (Figure 1).^18^ Thirty studies were included, and after citation searching was performed, 41 studies were included in the review, the characteristics of which are presented in Table S3. Some excluded studies examined outcomes of interest but lacked sufficient numbers of GPs (<5 early-career GPs).^24–26^ Studies examining training versus non-training practices without reporting career stage of the prescriber were excluded (*n* = 20).

Primary outcomes were investigated in 14 studies, 6 studies examined both primary and secondary outcomes and 21 studies investigated secondary outcomes. Over 30% of studies were from Europe (*n* = 15),^5,27–41^ 27% from Australia (*n* = 11),^42–52^ 24% from North America (*n* = 10),^6,53–58^ 10% from Asia (*n* = 4)^59–62^ and 1 study included multiple countries.^63^ Of studies examining primary outcomes (*n* = 20), more than half were from Australia (*n* = 11). Study designs were mostly either cohort (*n* = 21) or cross-sectional (*n* = 18). ORs, risk ratios and Pearson’s coefficient were commonly used to measure the association between antibiotic prescribing and experience of the GP.

### Primary outcomes

The primary outcomes, antibiotic prescribing rates, for any acute self-limiting respiratory condition ranged from 14.6%^57^ to 52%^51^ (Table 1). Antibiotic prescribing rates for URTI ranged from 13.5% in Australia^52^ to 29% in Canada,^21^ and for acute bronchitis ranged from 4.6% in Sweden^32^ to 63%–73% in Australia.^43,49,51^ Two studies^35,61^ included multiple acute self-limiting infections (RTIs, GITs, SSTIs, UTIs) and found that prescribing rates ranged from 11%^61^ to 26%.^35^

**Table 1.** Early-career GPs’ antibiotic prescribing rates for various acute infections

System/condition	Prescribing rate (%)	Country	Reference	Comments
Any acute infection (URTI, LRTI, GIT, SSTI, UTI)	25.7 14.6	Latvia	Likopa (2022)^35^	Experience 0–5 years Experience 6–10 years
Non-pneumonia respiratory tract infection and non-specific acute diarrhoea	Urban setting 33.2 Rural setting 24.1	Indonesia	Wardani (2021)^61^	Experience less than 7 years Individual condition data were not available
Any prolonged course of antibiotics^a^	30.5 ± 13.9 33.6 ± 18.3	Canada	Fernandez-Lazaro (2019)^19^	Experience 0–10 years Prescribing rate is proportion of total prescriptions that is prolonged
Respiratory				
* *Any respiratory condition^b^	52	Australia	Zwar (1994)^51^	GP registrars
* *Any acute respiratory condition^c^	24, *P* = 0.026	Malta	Saliba-Gustafsson (2019)^30^	<10 years in practice
* *Any acute respiratory condition^d^	23 (95% CI 22–24) 15 (95% CI 8–10)	Australia	Davey (2021)^46^	Immediate prescribing Delayed prescribing GP registrars^e^
* *Any respiratory condition^f^	14.9, *P* ≤ 0.001	USA	Walsh (2020)^57^	Family medicine resident physicians^g^
* *Acute bronchitis	83	Australia	Magin (2018)^50^	GP registrars
	74.6 (95% CI 73.4–75.8)	Australia	Baillie (2022)^52^	GP registrars
	73 (95% CI 70.4–75.9)	Australia	Dallas (2015)^43^	GP registrars
	72 (95% CI 69.6–74.6)	Australia	Magin (2016)^49^	GP registrars
	63	Australia	Zwar (1994)^51^	GP registrars
	26, *P* = 0.93	USA	Hueston (2000)^54^	Family medicine residents
	16	Sweden	Tell (2015)^32^	GP resident^h^
* *Cough	5	Sweden	Tell (2015)^32^	GP resident
* *URTI	33	Australia	Zwar (1994)^51^	GP registrars
	29 (IQR, 0.0–50.0)	Canada	Silverman (2017)^21^	10 years or less since graduation
	23	Hongkong	Dickinson (2002)^62^	Post-graduate doctors undergoing fellowship training Hong Kong College of Family Physicians or Diploma of Family Medicine
	22 (95% CI 20.1–23.1)	Australia	Dallas (2015)^43^	GP registrars
	13.5 (95% CI 13.2–14.0)	Australia	Baillie (2022)^52^	GP registrars
	16 (95% CI 14.9–17.8)	Australia	Magin (2016)^49^	GP registrars
	10.7		Magin (2018)^50^	
Ear, nose, throat				
* *Pharyngitis	59	Australia	Zwar (1994)^51^	GP registrars
* *Sinusitis	71 (95% CI 68.9–73.4)	Australia	Dallas (2017)^45^	GP registrars
	60	Australia	Zwar (1994)^51^	GP registrars
* *Acute otitis media	79 (95% CI 76.6–80.6)	Australia	Dallas (2017)^45^	GP registrars
	70	Australia	Zwar (1994)^51^	GP registrars
* *Sore throat	72 (95% CI 69.7–73.2)	Australia	Dallas (2016)^44^	GP registrars
* *Tonsillitis	84	Australia	Zwar (1994)^51^	GP registrars
Other systems				
* *Impetigo (systemic antibiotic)	59	Australia	Heal (2019)^48^	GP registrars
* *Impetigo (topical or systemic antibiotic)	94	Australia	Heal (2019)^48^	GP registrars
* *Conjunctivitis	74 (95% CI 72–76)	Australia	Cherry (2021)^42^	GP registrars
* *UTIs	86 (95% CI 84.7–87.2)	Australia	Davey (2020)^47^	GP registrars
* *Prolonged courses of antibiotics^i^ used for urinary infections	19.7 ± 14.4	Canada	Fernandez-Lazaro (2019)^19^	<11 years

^a^Prolonged antibiotic prescribing for respiratory drugs: penicillins, penicillins and β-lactamase inhibitor, cephalosporins, macrolides, extended-spectrum fluoroquinolones.

^b^Conditions: undifferentiated URTI, tonsillitis, streptococcal pharyngitis, sinusitis, acute bronchitis, otitis media.

^c^Conditions: LRTIs, URTIs, allergies and exacerbation of COPD/asthma/bronchitis.

^d^Conditions: pharyngitis, sore throat, URTI, acute bronchitis/bronchiolitis, acute sinusitis, acute otitis media, strep throat, acute tonsillitis).

^e^GP registrars: first 2 years in practice (Australia).

^f^Conditions: nasopharyngitis, acute laryngitis and tracheitis, acute laryngopharyngitis/upper acute respiratory infection, acute bronchitis, bronchitis not specified as acute or chronic, acute rhinosinusitis and acute pharyngitis.

^g^Family medicine resident: first 3 years in practice (USA).

^h^GP residents: first 5 years in practice (Sweden).

^i^Antibiotics: sulphonamides, trimethoprim, nitrofurantoin and fluoroquinolones.

Publications reporting prescribing rates for all other conditions were from Australia, using the same data source: the Registrars Clinical Encounters in Training (ReCEnT) study.^64,65^ The ReCEnT study is an ongoing inception cohort study in which GP registrars record 60 consecutive consultations every 6 months.^65^ Antibiotic prescribing rates were 59% for pharyngitis^51^, 71.5%–84% for sore throat/tonsillitis^44,51^ and 70%–78% for acute otitis media.^43,51^ Antibiotic prescribing rates were 59% for impetigo (systemic antibiotics),^48^ 74% for conjunctivitis^42^ and 86.4% for UTIs.^47^

### Secondary outcomes

Of the 27 studies examining secondary outcomes, 17 found a statistically significant relationship between experience and reduced prescribing, 8 found no statistically significant difference, and 2 found more experience resulted in less prescribing (Tables 2 and 3).

**Table 2.** Secondary outcomes—ORs comparing early-career GPs with later-career GPs on the prescription of antibiotics

Study ID	*n* ECGP/*n* GPs	Condition	Variable/reference/ comparator	Outcome, OR (95% CI)	Comments
Walsh (2020)^57^	62/415	Non-indicated conditions	Resident physician supervised by preceptor Attending (ref)	0.25 (0.18–0.36) 1 *P* ≤ 0.001	
Wardani (2021)^61^	8/16	Non-pneumonia RTI Non-specific diarrhoea	Work experience < 7 years (ref) 7 years or greater	1 Urban 3.194 (2.157–4.728), rural 3.779 (2.488–5.740)	
Cordoba (2015)^63^	32/52 AR 28/64 DK 20/28 LT 21/30 RU 63/257 SP 11/26 SW	Sore throat	Years as a practitioner 10 years or less >11 years (ref)	1.3 (0.2–2.6) AR 1.2 (0.4–3) DK 0.05 (0.01–0.3) LT 0.2 (0–42) RU 1.3 (0.7–2.3) SP 0.4 (0.04–3.6) SW 1	
Likopa (2022)^35^	NR/75	URTI, LRTI, SSTI, UTI	Working experience <5 years (ref) 6–10 years 11–20 years 20+ years	Univariable 1 0.49 (0.26–0.94) *P* = 0.03 1.32 (0.89–1.98) *P* = 0.17 1.39 (0.92–1.84) *P* = 0.13	Adjusted OR 1 0.70 (0.35–1.41) *P* = 0.32 1.52 (0.96–2.41) *P* = 0.08 1.28 (0.87–1.65) *P* = 0.21
Gjelstad (2009)^34^	NR/145	RTI	Year of medical exam 1991 or later 1981–1990 1971–1980 1958–1970 (ref)	0.89 (0.64–1.28) 0.62 (0.45–0.086) *P* = 0.57 0.90 (0.74–1.11) 1	GPs who sat their medical exam in the 1980s prescribed significantly fewer than those in the 1960s. Those who sat their exam even more recently found no correlation.
Saliba-Gustafsson (2019)^30^	6/30	Any antibiotic prescribed, indications not investigated	<10 years in practice >30 years in practice	1 1.77 (0.73–4.32) 2.81 (1.34–5.92) 3.05 (1.32–7.02) *P* = 0.026	ORs presented are from univariable model. Years in practice was removed from multivariable model and age of GP used due to collinearity: 28–39 years 1 40–49 years 1.45 (0.71–2.96) 50–59 years 2.12 (1.19–3.77) >60 years: 34.67 (14.14–84.98)
Fernandez-Lazaro (2019)^19^	10 616	Prescribing of prolonged courses of antibiotics	10 years or less (ref) 11–24 years >24 years	1 1.25 (1.16–1.34) 1.48 (1.38–1.58)	
Lo (2011)^60^	69/109	Any antibiotic prescribed, indications not investigated	Vocationally trained (VT) Non-vocationally trained (ref)	0.68 (0.63–0.74) *P* ≤ 0.05 1	VT trainees were 83.6% early career <11 years in practice
Steinke (2000)^31^	NR/231	Any antibiotic prescribed- indications not investigated	Training practices (ref) Non-training practices Inclusion of GP registrars in analyses	1 1.4 (1.39–1.43) 1.2 (1.18–1.21)	Including GP registrars’ data reduced the difference between training and non-training practices, indicating they prescribed closer to non-training practices. Article suggests it may be due to registrars seeing more patients requiring antibiotics.
Safaeian (2015)^59^	752/3772	Antibiotic classes, indications not investigated	Years since graduation <10 years Cephalosporins Macrolides Quinolones Aminoglycosides Penicillins Sulphonamides Tetracyclines >20 years (ref)	0.31 (0.24–0.41) 0.66 (0.51–0.84) 0.60 (0.50–0.75) 1.70 (1.23–2.37) 1.17 (0.90–1.52) 1.16 (1.10–1.41) 1.11 (1.01–1.31) 1	Sulphonamides, tetracyclines and penicillins were not significant at the *P* = 0.05 level.
Kitano (2020)^6^	NR/341	23 acute conditions	Years since medical graduation 0–10 years (ref) 11–24 years >24 years	1 0.96 (0.9–1.02) 1.04 (0.96–1.12)	Low prescribing GPs were removed from the study and number of GPs in each group not reported.
Schwartz (2019)^58^	NR/313	All patient encounters	Years since medical graduation 0–10 years (ref) 11–24 years >25 years	1 1 (0.88–1.14) 0.98 (0.84–1.13)	In their prescribing of all patient encounters: 0–10 years 6.22% 11–24 years 6.71% >25 years 6.29% *P* = 0.001

ECGP, early-career general practitioner; NR, not reported; AR, Argentina; DK, Denmark; LT, Lithuania; RU, Russia; SP, Spain; SW, Sweden.

**Table 3.** Measurements of the association between GPs’ working experience and antibiotic prescribing

Study ID	GPs (*n*)	Condition	Statistical measure	Variable	Outcome	Comments
Akkerman (2004)^39^	84	Ear, URTI, sinusitis, throat, pneumonia, bronchitis, exacerbation of COPD	Standardized coefficient	Years in practice	3.60 (1.20–6.0)	Years of practice was the most important factor in explaining the variation in prescribing antibiotics in this study.
Cadieux (2007)^41^	852	Viral respiratory condition	Risk ratio	Effect of each year in practice Prescribing for viral RTI Prescribing for second- or third-line antibiotics for bacterial infection	1.04 (1.02–1.05) 1.11 (CI 1.09–1.13)	
Pynnonen (2015)^56^	153	Acute sinusitis	OR	Years in practice Presence of a trainee during the consultation No trainee during consult (ref)	1.03 (0.99–1.07) 0.36 (0.2–0.65)1	
Silverman (2019)^21^	8990	Non-bacterial URTI	Percentage-point difference	<11 years 11–24 years	1 5.1 (3.9–6.4), *P* < 0.001	
Kuyvenhoven (1993)^28^	161	URTIs, AOM, acute sinusitis, acute tonsillitis	Pearson’s R	Years since settlement	−0.29, *P* < 0.05	Those more recently settled prescribed fewer
Mainous (1998)^55^	205	URTI, acute nasopharyngitis, common cold	*t*-test	Time since graduation Low prescribers High prescribers	19.5 years ±1.06 26.9 years ±10.9 *P* < 0.001	Low prescribers had fewer years since medical school
Veninga (2000)^40^	562	Uncomplicated UTI	Increase in explained variance of guideline adherence	Fewer years in practice Norway Sweden Across countries	11% variance in first choice drugs 6% variance in duration of treatment 1% variance in duration of treatment	Selecting first-choice drugs was related to being in practice for fewer years in Norway In Sweden, GPs with fewer years in practice tended to prescribe shorter treatments
Nicolle (2012)^37^	2346	Any antibiotic prescription	DDDs (no statistical measures)	Seniority years <5 years 5–19 years 20–29 years >30 years	1.63 6.80 6.51 2.95	
De Sutter (2001)^5^	80	Sinusitis	Pearson’s R	Number of years in practice	−0.028, *P* = 0.83	Not statistically significant
Gill (2001)^33^	155	Any antibiotic	Multivariable analysis	Length of time in general practice	Reported as non-significant variable in analysis	Not statistically significant
Martinez-Gonzalez (2020)^36^	240	Any systemic antibiotic	Univariable regression analysis	Years in practice	0.006 (−0.0004 to 0.012) *P* = 0.068	
Petrovic (2019)^29^	200	Acute bronchitis	Mann–Whitney *U* test	Working experience	*U* = 3369.0 *P* = 0.985	
Di Martino (2017)^27^	4323	Any antibiotic prescribed to a paediatric patient	ORs	Years of experience in 5-year increments Age 0–5 years Age 6–13 years	0.92 (0.89–0.95) 0.97 (0.96–0.99)	Paediatricians’ prescribing (prescribers not applicable to this review) 85% were GPs, with 15% paediatricians, paediatricians on average prescribed lower (approx. 5%) each year than GPs.

AOM, acute otitis media.

### Studies concluding less-experienced GPs prescribed fewer antibiotics

Of the studies with a statistically significant relationship (*n* = 17), 7 found that early-career GPs have decreased odds of prescribing antibiotics compared with later-career GPs, with OR ranging from 0.25 to 0.68.^19,30,31,35,57,60,61^ The other 10 found that working experience significantly influenced antibiotic prescribing. Walsh *et al*.,^57^ examining antibiotic prescribing for ‘non-indicated conditions’, reported the lowest OR of 0.25 (95% CI 0.18–0.36).^57^ Akkerman *et al*.^39^ concluded that ‘years in practice’ was the most important factor explaining variation in antibiotic prescribing, accounting for 29% of prescribing variability.^39^ Mainous *et al*.^55^ found that lower antibiotic prescribers (25^th^ percentile and below) compared with high prescribers (75^th^ percentile and above) had significantly fewer years since graduation.^55^

Two studies found partially significant results, depending on the country or the antibiotic class prescribed.^59,63^ Safaeian *et al*.^59^ examined 3372 GPs’ prescribing of different antibiotic classes, and found that early-career GPs were less likely to prescribe cephalosporins, macrolides and quinolones, but more likely to prescribe an aminoglycoside. Neither study found a statistically significance difference for penicillins, sulphonamides and tetracyclines, compared with later-career GPs.^59^

Cordoba *et al*.^63^ examined prescribing for sore throat across six countries; Lithuania was the only country with a statistically significant relationship between years in practice (OR 0.05; 95% CI 0.01–0.3).^63^ However, all countries had very low sample sizes of early-career GPs (11–63), overall small sample sizes and high variability in prescribing between GPs.^63^

### Studies that did not identify a relationship between experience and antibiotic prescribing

Of the eight studies that found no statistically significant relationship, three compared early-career with late-career GPs’ antibiotic prescribing,^6,34,58^ and five examined the influence of years in practice on antibiotic prescribing.^5,29,33,36,66^ Seven of the eight studies did not report the number of early-career GPs included, or the range of years in practice. The one study that did report this included eight GPs with 5–10 years’ experience, and no GPs with experience of <5 years.^5^

Two studies provided additional information regarding GPs in training.^29,56^ Pynnonen *et al*.^56^ found that ‘having a GP trainee present during a patient visit’ reduced the likelihood of prescribing (OR 0.36; 95% CI 0.2–0.65).^56^ In the study by Petrovic *et al*.,^29^ physicians with specialist training in general practice had a lower likelihood of prescribing (OR 0.35; 95% CI 0.15–0.82, *P* = 0.016) compared with those who practice without specialist training (in Serbia, one can practice as a GP without post-graduate GP training).^29^

### Studies finding more-experienced GPs prescribed fewer antibiotics

Di Martino *et al*.^27^ found that, with an increase in increments of 5 years’ experience, the odds of prescribing reduced (OR 0.97; 95% CI 0.96–0.99).^27^ They examined all patients aged 6–13 years in a region of Italy, including 5097 physicians, 15% of which were paeditricians.^27^

Degnan *et al*.^53^ found that prescribers who were board-certified before 1997 had a lower rate of antibiotic prescribing compared with those registered more recently (63% versus 76%, *P* = 0.02). Those in teaching practices in this study prescribed 22% fewer antibiotics (73% versus 51%, *P* ≤ 0.01).^53^

### Risk of bias

The 39 observational studies were generally considered at low risk of bias, with only 3 having serious risk of bias using the Newcastle–Ottawa Scale (Table 4). The main concerns were sample size (*n* = 10), selection bias (*n* = 8) or confounding (*n* = 10). Selection bias was due to either: not being representative of GPs in their country (*n* = 6); excluding low antibiotic prescribing GPs (*n* = 1); or GPs were aware of the study aims (*n* = 1). The majority of records controlled both patient and GP factors, although some may have been subject to confounding, either by only focusing on GP factors/not controlling patient factors, or by controlling only a small number of confounders. Outcome measurement across almost all studies was appropriate, with most using record-linked data (*n* = 37; 95%).

**Table 4.** Risk-of-bias assessment of included studies, using the Newcastle–Ottawa Scale adapted for cross-sectional, cohort and case–control studies, and the Cochrane Risk of Bias tool for RCTs

Study (year)	Selection	Comparability	Outcome	Total	Overall
Cross-sectional					
* *Baillie (2022)	4	2	3	9	Good
* *Cherry (2021)	4	2	3	9	Good
* *Cordoba (2015)	2	2	3	7	Fair
* *Dallas (2015)	4	2	3	9	Good
* *Dallas (2016)	4	2	3	9	Good
* *Dallas (2017)	4	2	3	9	Good
* *Davey (2020)	4	2	3	9	Good
* *Davey (2021)	4	2	3	9	Good
* *De Sutter (2001)	1	1	3	4	Poor
* *Di Martino (2017)	3	1	3	7	Good
* *Dickinson (2002)	4	0	3	7	Good
* *Gill (2001)	4	2	3	9	Good
* *Heal (2019)	4	2	3	9	Good
* *Hueston (2000)	3	1	3	7	Good
* *Kuyvenhoven (1993)	2	2	3	7	Fair
* *Magin (2016)	4	2	3	9	Good
* *Mainous (1998)	3	2	3	8	Good
* *Martinez-Gonzalez (2020)	4	2	3	9	Good
* *Safaeian (2015)	4	1	3	8	Good
* *Saliba-Gustafsson (2019)	1	2	3	6	Poor
* *Silverman (2017)	4	2	3	9	Good
* *Tell (2015)	4	1	3	8	Good
* *Veninga (2000)	4	1	3	8	Good
* *Walsh (2020)	4	2	3	9	Good
* *Zwar (1994)	4	2	2	8	Good
Case–control					
* *Petrović (2019)	4	1	3	8	Good
Cohort studies					
* *Akkerman (2004)	3	2	3	8	Good
* *Cadieux (2007)	4	1	3	8	Good
* *Degnan (2021)	4	2	3	9	Good
* *Fernandez-Lazaro (2019)	4	2	3	9	Good
* *Gjelstad (2009)	4	2	3	9	Good
* *Kitano (2020)	3	2	3	8	Good
* *Lo (2011)	3	2	3	8	Good
* *Nicole (2012)	3	2	3	8	Good
* *Pynnonen (2015)	3	2	3	8	Good
* *Schwartz (2019)	4	2	3	9	Good
* *Steinke (2000)	3	2	3	8	Good
* *van Duijn (2007)	4	1	3	8	Good
* *Wardani (2021)	1	0	3	4	Poor

Intervention studies	Selection bias	Performance bias	Detection bias	Reporting bias	Attrition bias	Overall
* *Likopa (2022)	High	High	High	Unclear	High	High
* *Magin (2018)	Low	Unclear	Low	Unclear	Low	Low/unclear

One of the two RCTs had a high risk of bias; participants were aware of their allocation and 5/40 GPs in the control group dropped out after randomization.^35^ Reason for declining to participate was not reported; however, participants may have declined after randomization as they would not receive the C-reactive protein testing kits given to the intervention group (not readily available in Latvian general practice).^35^ Participants recorded their own prescribing, and were not required to record all consultations for infections. The other RCT had a lower risk of bias; although it did not report if participants were aware of the intervention, the intervention was embedded in their regular GP training (unlikely to cause performance bias).^50^

## Discussion

In the majority of studies identified, early-career GPs prescribed fewer antibiotics than later-career GPs, across a variety of conditions and countries. Although highly heterogeneous in variables used to describe the outcome and measurement of the variables, most studies found more years in practice was associated with higher likelihood of antibiotics prescribed. This may be encouraging for future antibiotic stewardship if these lower antibiotic prescribing rates of newer generations of GPs reflect increased awareness of the importance of antimicrobial resistance over the past decade.^2,67^ However, it is also reasonable to speculate that GPs begin their career with more evidence-based prescribing, but this may deteriorate with time in practice, due to financial, time and patient pressures.^68^ One of the included studies suggests this may be the case; Cadieux *et al*.^41^ found that the effect of 5 years in practice was associated with increased antibiotic prescribing (OR 1.11; 95% CI 1.09–1.13). Furthermore, for most conditions, although early-career GPs’ prescribing is lower compared with more experienced peers, antibiotics continue to be overprescribed.

### Strengths and limitations

We identified studies from different continents and across different health systems. However, nearly a third of studies included in our review are from the Australian ReCEnT study. This is both a strength and limitation. The ReCEnT study records in-depth real-time information of GP trainee clinical encounters with patients, across a comprehensive range of geographical and socioeconomic Australian settings, and has limited biases.^49^ However, ReCEnT captures data only in the first 2 years of clinical experience in vocational training and we do not know if prescribing habits persist. In addition, it makes our review Australia-centric, particularly with regard to prescribing rates, limiting generalizability of the findings.

The search strategy was narrowed to include early-career GP terminology: this may have excluded studies from a wider range of GP career stages that didn’t separately present data of early-career GPs. There may also be other terms for GPs in training used in non-English-speaking countries that were not identified in our search string for ‘early-career’. To mitigate this potential selection bias, a comprehensive citation search was performed.

Most of the literature was current (70% from the past 10 years); however, three of the studies were published prior to 2000.^28,51,55^ Two of these found more-experienced GPs prescribed more, and the other reported prescribing rate data.^28,55^ Some of the included studies had small sample sizes, or were from single regions, which may not be representative of GPs in their respective countries. Many studies examined the association between prescriber age and antibiotic prescribing but were excluded as working experience may vary across GP ages.

We intended to perform meta-analysis, but this was not appropriate due to methodological and clinical heterogeneity of the included studies. The PROSPERO protocol stated that secondary outcomes were ‘appropriateness of antibiotic (first line, second line, specified with regard to authoritative prescribing guideline in country)’. This was changed, because first-line antibiotic treatment choice differs across international guidelines, and therefore was not comparable. This was revised to be ‘any associations between working experience of the GP and antibiotic prescribing’, after the search was performed, but before full-text screening. The change of the secondary outcome was needed and added important information to the narrative about antibiotic prescribing of early-career GPs.

### Comparison with existing literature

A systematic review by Hawkins *et al*.^69^ comparing Australia, Sweden and the UK found that neither antibiotic consumption nor community knowledge has changed significantly in Australia and the UK since 2011. In line with the Hawkins review,^69^ we also found the lowest antibiotic prescribing rates in Sweden, a country with established low antibiotic prescribing.^70^

A previous qualitative review determined that key driving factors of unnecessary prescribing included diagnostic uncertainty, time pressure and patient pressure.^68^ A qualitative study of GPs in vocational training presented similar themes, but also pointed to the inexperience of the GP, and the influence of the supervisor.^15^ GPs in training viewed guidelines favourably, and following them was deemed desirable.^15^ Conversely, in the literature review, lack of adherence to guidelines/continuing professional education was noted by 10 of the 17 studies.^68^ Less-experienced GPs’ preference to use guidelines, in addition to their recent medical education, may explain why their prescribing is lower than more-experienced GPs.^15,68^

Many of the excluded papers examined training versus non-training practices, much of which suggest that status as a training practice is associated with lower prescribing.^71–73^ Although data from training practices includes early-career GPs, supervisors who may be of varying experience were also included in ‘training practices’ as their unit of analysis. Training practices’ antibiotic prescribing may be of interest for further review, as knowledge in this area could inform medical education. Data on early-career GPs, particularly after vocational/specialist training, are still lacking.

### Implications for research and/or practice

We found limited international data on early-career GPs’ antibiotic prescribing, and this varied by country. Antimicrobials continue to be overprescribed, even by early-career GPs, who have had recent medical education. Antimicrobial resistance is a global problem, and it is important to achieve a greater understanding of early-career GPs’ prescribing in a wider range of settings. A previous non-randomized trial of education targeting GP trainees demonstrated a (short-term) substantive decrease in antibiotic prescribing for acute bronchitis.^50^ Further interventions targeting early-career GPs could examine effects on antibiotic prescribing for other conditions (and assess longer-term sustained changes), so contributing to future antimicrobial stewardship.

## Supplementary Material

dkae002_Supplementary_Data

## Supplementary data

Tables S1–S3 are available as Supplementary data at *JAC* Online.
