# Supplementary material for: Early-career general practitioners’ antibiotic prescribing for acute infections: a systematic review
Source: J Antimicrob Chemother. 2024 Jan 22;79(3):512–25. doi: 10.1093/jac/dkae002 (PMC10904722; doi:10.1093/jac/dkae002)
Supplement: dkae002_Supplementary_Data [file dkae002_supplementary_data.docx]

Table S1: Search strategy

| **Pubmed** |
| --- |
| ((((((((trainee[Title/Abstract]) OR (student[Title/Abstract])) OR (training[Title/Abstract])) OR (vocation[Title/Abstract])) OR (vocational[Title/Abstract])) OR (resident[Title/Abstract])) OR (early career[Title/Abstract])) OR (registrar[Title/Abstract])) AND (((((general practice[Title/Abstract]) OR (general practitioner[Title/Abstract])) OR (family medicine[Title/Abstract])) OR (community care[Title/Abstract])) OR (primary care[Title/Abstract]))) AND (((((((antibiotic[Title/Abstract]) OR (antibiotics[Title/Abstract])) OR (antibacterial agent[Title/Abstract])) OR (antiinfective agent[Title/Abstract])) OR (antimicrobials[Title/Abstract])) OR (resistant[Title/Abstract])) OR (resistance[Title/Abstract]))  Results: 493 |
| **Embase** |
| ('antibiotic agent':ti,ab,kw OR 'antiinfective agent':ti,ab,kw OR antimicrobials:ti,ab,kw OR resistance:ti,ab,kw OR resistant:ti,ab,kw OR antibiotics:ti,ab,kw) AND ('student':ti,ab,kw OR student:ti,ab,kw OR trainee:ti,ab,kw OR (early:ti,ab,kw AND career:ti,ab,kw) OR registrar:ti,ab,kw OR vocation:ti,ab,kw OR resident:ti,ab,kw OR training:ti,ab,kw) AND ('primary medical care':ti,ab,kw OR 'general practitioner':ti,ab,kw OR 'general practice':ti,ab,kw OR 'community care':ti,ab,kw OR 'family medicine':ti,ab,kw OR gp:ti,ab,kw)  Results: 391 |
| **Scopus** |
| ( TITLE-ABS-KEY ( "primary care" OR "general practice" OR "general practitioner" OR "community care" OR "family medicine" OR "family practice" OR "general practitioner" ) AND TITLE-ABS-KEY ( "early career" OR "trainee" OR "registrar" OR "resident" OR "vocational" OR "student" ) AND TITLE-ABS-KEY ( "antibiotic" OR "anti-bacterial agent" OR "resistance" OR "anti-infective agent" OR "antimicrobial stewardship" OR "antibiotics" OR "resistant" ) )  Results: 609 |

*Table S2: Risk of Bias domains of interest, Newcastle-Ottowa Scale*

|  | **Selection**  (Maximum 4 stars) | | | | **Comparability**  (Maximum 2 stars) | **Outcome**  (Maximum 3 stars) | | |
| --- | --- | --- | --- | --- | --- | --- | --- | --- |
| **Case-control** | 1. Representativeness of the exposed cohort | 2. Sample Size | 3. Non-respondents | 4. Ascertainment of the exposure (absence or exclusion) | The subjects in different outcome groups are comparable, based on the study design or analysis. Confounding factors are controlled | 1. Assessment of outcome | 2. Statistical test |  |
| **Cohort studies** | 1. Representativeness of the exposed cohort | 2. Selection of the non-exposed cohort | 3. Ascertainment of exposure | 4. Demonstration that outcome of interest was not present at start of study | Comparability of cohorts on the basis of the design or analysis controlled for confounders | 1. Assessment of outcome | 2. Was follow-up long enough for outcomes to occur? | 3. Adequacy of follow-up of cohorts |
| **Cross-sectional studies** | 1. Representativeness of the exposed cohort | 2. Sample Size | 3. Non-respondents | 4. Ascertainment of the exposure (absence or exclusion) | The subjects in different outcome groups are comparable, based on the study design or analysis. Confounding factors are controlled | 1. Assessment of outcome | 2. Statistical test |  |

Table S3: Characteristics of studies included in the review

| **Author (Year)** | **Country** | **Design** | **(n) GPs** | **Measure of 'early career' or working experience variable** | **Average age of GP (years)** | **Sex n, (%)** | **Outcomes examined** |
| --- | --- | --- | --- | --- | --- | --- | --- |
| **Akkerman (2004)^32^** | The Netherlands | Descriptive cohort study | 84 | Working experience 0-10 years | NR | 57 Male (68%) | Secondary |
| **Baillie (2022)^44^** | Australia | cross-sectional analysis of an ongoing prospective cohort study. | 2,839 | Registrars, first 18 months in general practice | 32.6 (6.3) | 1,762 Female (62.5%) | Primary |
| **Cadieux (2007)^60^** | Canada | longitudinal study design | 852 | First 6-9 years in practice | NR | 366 Male (43%) | Secondary |
| **Cherry (2021)^34^** | Australia | cross-sectional analysis of an ongoing prospective cohort study. | 2,333 | Registrars, first 18 months in general practice | 32 (6.3) | 1467 (62.9%) | Primary |
| **Cordoba (2015)^61^** | Argentina  Denmark  Lithuania  Russia  Spain  Sweden | Cross-sectional study | 52 AR  64 DK  28 LT  30 RU  257 SP  26 SW  Total: 175/457 (38%) | Years working as a GP <10 years | NR | 32 (69%) AR  31 (69%) DK  24 (85%) LT  26 (86%) RU  164 (64%) SP  9 (34%) SW  Total: 290 (63%) | Secondary |
| **Dallas (2015)^35^** | Australia | cross-sectional analysis of an ongoing prospective cohort study. | 401 | Registrars, first 18 months in general practice | 33.26 (6.73) | 275 (68.6%) | Primary |
| **Dallas (2016)^36^** | Australia | cross-sectional analysis of an ongoing prospective cohort study. | 856 | Registrars, first 18 months in general practice | 32.5 (6.3) | 562 (65.7%) | Primary |
| **Dallas (2017)^37^** | Australia | cross-sectional analysis of an ongoing prospective cohort study. | 856 | Registrars, first 18 months in general practice | 32.5 (6.3) | 562 (65.7%) | Primary |
| **Davey (2020)^39^** | Australia | cross-sectional analysis of an ongoing prospective cohort study. | 1,333 | Registrars, first 18 months in general practice | 32.6 (6.3) | (34.7%) Male | Primary |
| **Davey (2021)^38^** | Australia | cross-sectional analysis of an ongoing prospective cohort study. | 631 | Registrars, first 18 months in general practice | 32.5 (6.3) | 384 (61%) | Primary |
| **Degnan (2021)^45^** | USA | retrospective cohort study | 60 | various | NR | 37 (62%) | Secondary |
| **De Sutter (2001)^19^** | Belgium | Cross-sectional observational | 80 | No. years in practice | NR | 4 (5%) | Secondary |
| **Di Martino (2017)^20^** | Italy | retrospective observational study | 4,323 | Years of experience | NR | NR | Secondary |
| **Dickinson (2002)^56^** | Hong Kong | Diagnosis and drug data obtained from logbooks | 144 | Postgraduate family medicine training | NR | NR | Primary |
| **Fernandez-Lazaro (2019)^14^** | Canada | retrospective cohort analysis | 10,616 | Less than 11 years in practice | NR | 4694 (44.2%) | Primary and secondary |
| **Gill (2001)^26^** | UK (England) | Secondary analysis of cross-sectional data | 155 | Length of time in general practice | 47.3 (7.9) | 6:4:1 | Secondary |
| **Gjelstad (2009)^27^** | Norway | Prospective cohort study | 145 | Year of medical exam | NR | NR | Secondary |
| **Heal (2019)^40^** | Australia | cross-sectional analysis of an ongoing prospective cohort study. | 1,741 | Registrars, first 18 months in general practice | 33(7) | 232(57%) | Primary |
| **Hueston (2000)^46^** | USA | retrospective chart review | 51 | Family practice resident physicians/resident | NR | NR | Primary |
| **Kitano (2020)^50^** | Canada | Retrospective cohort study | 341 | Years since medical graduation | 44.5 | 194 (56.9%) | Secondary |
| **Kuyvenhoven (1993)^21^** | The Netherlands | analysis of dutch national survey | 161 | Years since settlement | NR | NR | Secondary |
| **Likopa (2022)^28^** | Latvia | control arm of a randomised controlled trial | 35 | Work experience <5 years and 6-10 years | 53 (median age, IQR 46-61) | 34 (97.1%) | Primary and secondary |
| **Lo (2011)^54^** | Hong Kong | prospective observational study | 109 | Less than 10 years in general practice | 42.4 (+/- 14.2) | 36 (33.0%) | Secondary |
| **Magin (2016)^41^** | Australia | cross-sectional analysis of an ongoing prospective cohort study. | 856 | Registrars, first 18 months in general practice | 32.5 (+/-6.3) | 562 (65.7%) | Primary |
| **Magin (2018)^42^** | Australia | control arm of a non-randomised controlled trial | 311 | Registrars, first 18 months in general practice | 31.1 (+/-5.2) | 204 (66%) | Primary |
| **Mainous (1998)^47^** | USA | retrospective analysis of episodes of care | 205 | Time since medical school graduation | 46.4+/-10.4 | 47(85%) | Secondary |
| **Martinez-Gonzalez (2020)^29^** | Switzerland | Retrospective cross-sectional study | 240 | Years in practice | NR | NR | Secondary |
| **Nicolle (2012)^30^** | France | Retrospective cohort study | 2,346 | Years in practice (“seniority”) | 53.5 | 614 (26.2%) | Secondary |
| **Petrovic (2018)^22^** | Serbia and Montenegro | observational case/control study | NR* | Working experience of the physician | 49.8 +/- 6.9 [AB]  49.6 +/- 6.4 [No AB] | 152 (96.8%) [AB] 42 (97.7%) [No AB] | Secondary |
| **Pynnonen (2015)^48^** | USA | Retrospective cohort | 153 | Years in practice | NR | 81(52.9%) | Secondary |
| **Safaeian (2015)^52^** | Iran | Restrospective cross-sectional study | 3,772 | Less than 10 years since graduation | NR | 960 (25.5%) | Secondary |
| **Saliba-Gustafsson (2019)^23^** | Malta | cross-sectional surveillance study | 30 | Less than 10 years of practice | 49 (+/-12) | 24 (73%) male | Primary and secondary |
| **Schwartz (2019)^51^** | Canada | Cohort study | 313 | 10 years or less since graduation | NR | 177 (56.6%) | Secondary |
| **Silverman (2017)^16^** | Canada | retrospective analysis of linked administrative data | 8,990 | 10 years or less since graduation | NR | 3705 (41.2%) | Primary |
| **Steinke (2000)^24^** | UK (Scotland) | cohort study using record linked database | 231 | Registrars, first 18 months in general practice | NR | NR | Secondary |
| **Tell (2015)^25^** | Sweden | retrospective study of structured data from electronic patient records | 753 | Family medicine residents, first 5 years in practice | NR | NR | Primary |
| **van Duijn (2005)^31^** | The Netherlands | Retrospective cohort study | 163 | Years of practice | 47.1 (95% CI 46.1-48.1) | 26.4% | Secondary |
| **Veninga (2000)^33^** | Sweden  The Netherlands  Norway | Cross-sectional study | 562 | Years in practice | NR | NR | Secondary |
| **Walsh (2020)^49^** | USA | retrospective analysis using electronic health records | 415 | Primary care residents, first 3 years in practice | NR | NR | Primary and secondary |
| **Wardani (2021)^55^** | Indonesia | Retrospective cohort study | 16 | Experience less than 7 years | NR | 15 (93.8%) | Primary and secondary |
| **Zwar (1994)^43^** | Australia | cross sectional analysis | 46 | Registrars, first 18 months in general practice | 27.6 | 22 (48%) | Primary |
| ***NR= not reported** | | | | | | |  |
